# Porcine Macrophage Markers and Populations: An Update

**DOI:** 10.3390/cells12162103

**Published:** 2023-08-19

**Authors:** Belén Álvarez, Concepción Revilla, Teresa Poderoso, Angel Ezquerra, Javier Domínguez

**Affiliations:** Departamento de Biotecnología, CSIC INIA, Ctra. De La Coruña, km7.5, 28040 Madrid, Spain; avega@inia.csic.es (B.Á.); crevilla@inia.csic.es (C.R.); poderoso.teresa@inia.csic.es (T.P.); juncal@inia.csic.es (J.D.)

**Keywords:** swine, macrophage, surface markers, phenotype, subsets

## Abstract

Besides its importance as a livestock species, pig is increasingly being used as an animal model for biomedical research. Macrophages play critical roles in immunity to pathogens, tissue development, homeostasis and tissue repair. These cells are also primary targets for replication of viruses such as African swine fever virus, classical swine fever virus, and porcine respiratory and reproductive syndrome virus, which can cause huge economic losses to the pig industry. In this article, we review the current status of knowledge on porcine macrophages, starting by reviewing the markers available for their phenotypical characterization and following with the characteristics of the main macrophage populations described in different organs, as well as the effect of polarization conditions on their phenotype and function. We will also review available cell lines suitable for studies on the biology of porcine macrophages and their interaction with pathogens.

## 1. Introduction

Pig is an economically important livestock species, accounting for about one-third of all meat production worldwide (Food and Agriculture Organization of the United Nations: https://www.fao.org/faostat/en/#data/QCL (accessed on 13 June 2023)). It is also increasingly used as an animal model in biomedical studies on cardiovascular, infectious, cancer and nutritional diseases because of some similarities to humans in anatomical size and structure, physiology, and genome [1,2].

Macrophages are long-lived professional phagocytic cells distributed in all organs through the body, where they perform a broad range of functions. In addition to providing a first line of defense against microbial pathogens, engulfing and killing them and producing inflammatory cytokines that recruit other immune cells to the site of infection, they play important roles in organogenesis and tissue homeostasis, removing cell debris and potentially harmful agents, promoting wound healing and controlling inflammation after injury, and regulating tissue metabolism [3,4,5].

Several viruses, such as African swine fever virus (ASFV), classical swine fever virus (CSFV) and porcine reproductive and respiratory syndrome virus (PRRSV), which stand out among major threats that concern the pig industry, use macrophages as their main target for replication [6,7]. These cells also play a crucial role in the pathogenesis of processes with an important inflammatory component, such as obesity, diabetes, cancer, atherosclerosis, or myocardial infarction, for which pigs are increasingly being used as the model for translational research [2].

Therefore, improvements in our knowledge of the biology and function of porcine macrophages, and their interaction with pathogens, will contribute to a better understanding of the mechanisms underlying the pathogenesis of the aforementioned diseases and to devising effective strategies to fight them. 

This article is a classical review in which a literature review of covered issues was routinely but not exhaustively performed over time. We review current knowledge on markers most frequently used for phenotypical characterization of porcine macrophages. Since the publication in 2009 of our review on porcine myelomonocytic markers and cell populations [8], there have been significant advances in the molecular characterization of new cell surface receptors expressed on porcine macrophages and the dissection of macrophage populations residing in different organs, as well as in methods for the development of immortalized cell lines very useful for studies on the biology of these cells and their interaction with pathogens.

## 2. Markers for Studying Monocytes/Macrophages and Their Differentiation in Swine

Macrophages express a broad array of surface receptors that enables them to sense the presence of pathogens and changes in their local microenvironment and carry out the great variety of functions that they can perform.

Although tools for the study of porcine immune system lie far behind those available for humans and rodents, the field has experienced considerable progress in recent years, and reagents for many surface receptors commonly used in the characterization of monocyte/macrophage populations are now available for swine (Table 1). Here, we will revise markers commonly used in the characterization of porcine monocyte/macrophages. A more exhaustive list of available reagents for immunological analyses in the pig can be found in [9].

### 2.1. CD172a

Porcine CD172a was originally clustered as SWC3 at the First International Swine CD Workshop, and it has since been widely used for the identification of myeloid cells in swine [21,38]. It is found in early precursors of the myelomonocytic lineage, being expressed at high levels in macrophages, monocytes and granulocytes and at lower levels on dendritic cell (DC) subsets [39,40,41]. It is also expressed at low levels on bone marrow B cell precursors, but this expression is lost in more differentiated stages of this lineage [42].

CD172a (SIRP alpha) is the prototypic member of the signal-regulatory protein (SIRP) family of paired receptors. Structurally, porcine CD172a is a type I membrane protein. As such, it has three differentiated segments, an extracellular region, a transmembrane segment and a cytoplasmic tail. The first is composed of three immunoglobulin (Ig)-like domains (an N-terminal V-type domain, and two C1-type domains). The cytoplasmic tail contains immunoreceptor tyrosine-based inhibitory motifs (ITIMs) [43]. A splicing variant of this molecule has been described which only contains the V-type domain in the extracellular region, not showing other differences with the prototype molecule, and which is also expressed in pulmonary alveolar macrophages (PAM) [43].

CD172a interacts with CD47, a membrane protein with a broad tissue distribution, through its amino-terminal Ig-like domain. CD47 serves as a “marker of self”; upon binding of CD47 to CD172a, ITIM motifs in the cytoplasmic region of CD172a obtain their tyrosine residues phosphorylated and recruit SHP-1 and SHP-2 tyrosine phosphatases that negatively regulate signal transduction cascades, preventing phagocytosis [44]. Whereas porcine CD172a can bind to human CD47, resulting in a blockade of phagocytosis, which facilitates the engraftment of human hematopoietic stem cells into severe combined immunodeficient (SCID) pigs for the development of humanized animals [45], the failure of porcine CD47 to bind to human CD172a may contribute to the rejection of xenogeneic target cells by a recipient’s macrophages [46]. 

CD172a has also been shown to interact with surfactant proteins SP-A and SP-D, contributing to the control of inflammatory responses in the lung [47].

In humans and rodents, the SIRP family comprises several members, encoded by different genes. The ectodomains of these proteins are highly homologous in their sequences, while the transmembrane and cytoplasmic regions are very different or absent. On the basis of such differences, SIRP proteins are classified in four groups (α, β, γ and δ), CD172a belonging to the α group [48]. There are no available data on the expression on porcine cells of other members of the SIRP family, such as the closely related SIRP β1, which could provide an activating signal; although, at least a *sirpb1* gene has been identified in the swine genome. 

### 2.2. CD14

CD14 acts as a co-receptor, along with TLR4 and MD-2, for the detection of lipopolysaccharides (LPS) of Gram-negative bacteria [49,50]. The binding of LPS to CD14 is more efficient when LPS is bound to a serum protein called LPS-binding protein (LBP) [51]. CD14 not only recognizes LPS, as it also interacts with other pathogen-associated molecular patterns, such as lipoteichoic acid and peptidoglycans from Gram-positive bacteria, glycolipids from mycobacteria and mannans from yeasts [52,53,54,55]. The interaction of CD14 with ligands signalizes through associated TLRs, which results in the expression of co-stimulatory receptors and the production of cytokines with pro-inflammatory properties [54,55]. Soluble forms of CD14 are present in a variety of body fluids [56,57] and have been shown to enhance the binding of triacylated lipopeptides to TLR1 and TLR2 and drive the formation of TLR1/TLR2 heterodimers [58].

Porcine CD14 is a glycophosphatidyl inositol (GPI)-anchored membrane glycoprotein with an extracellular domain containing 11 leucine-rich repeats [59,60]. Characterization of this marker in pig relied initially on the use of anti-human CD14 cross-reactive monoclonal antibodies (mAbs), but specific antibodies to porcine CD14 have also been developed [61,62,63]. It is expressed on monocytes, tissue macrophages and, at lower levels, granulocytes. Differences in staining patterns among distinct CD14-specific antibodies have been observed. For instance, anti-human cross-reactive biG10 and TÜK4 mAbs stain low percentages of PAM and granulocytes, whereas the porcine-specific mAb MIL2 and anti-human cross-reactive mAb MY4 stain most of these cells and show stronger labelling of monocytes [61,63,64]. Dual labelling and competitive blocking experiments suggest that these mAbs recognize the same antigen but with different affinities, although recognition of different conformations of the molecule cannot be excluded [63,64].

A soluble form of CD14, produced by shedding from the cell surface, has been identified in porcine serum and milk [61,65].

### 2.3. Fc-Gamma Receptors

IgG Fc receptor (FcγR) family comprise both activating and inhibitory receptors, encoded by different genes, which are expressed in macrophages and other immune cells, and are involved in a wide variety of processes, such as phagocytosis, antibody-dependent cellular cytotoxicity (ADCC), regulation of cytokine production, macrophage polarization, and antigen internalization and presentation [66,67,68]. In the pig, four members of this family have been identified: FcγRI (CD64), FcγRIIa (CD32A), FcγRIIb (CD32B), and FcγRIIIa (CD16) [13,14,69,70]. The genomic organization of genes that encode these molecules in the pig is like that of other mammals; however, no genes coding for homologs of the human FcγRIIc and FcγRIIIb have been identified [14].

FcγRI (CD64) is a high-affinity receptor, which can bind monomeric IgG as well as aggregated IgG, whereas FcγRII (CD32) and FcγRIIIa (CD16) are low-affinity receptors that only bind aggregated IgG or IgG in immune complexes. The binding affinity differs depending on IgG subclasses [71]. While FcγRI (CD64), FcγRIIa (CD32A), and FcγRIIIa (CD16) are activating receptors, FcγRIIb (CD32B) is an inhibitory receptor that can counteract the effects mediated by the activating receptors [66].

Porcine FcγRI (CD64) is made up of three extracellular Ig-like domains, a transmembrane segment, and a short cytosolic tail with no known signaling motifs [70]. In human and mouse, FcγRI (CD64) associates with the Fc receptor common gamma chain (FcRγ) homodimer for its signal transduction activity [66].

Porcine FcγRIIa (CD32A) and FcγRIIb (CD32B) have a high amino-acid (aa) sequence homology (>90%) in their two Ig-like extracellular domains but differ in their cytosolic region. Porcine FcγRIIa, like its bovine and murine homologs, in contrast to human FcγRIIa, lacks an ITAM motif in its cytosolic tail, and requires interaction with FcRγ chain for signaling [14]. Porcine FcγRIIb possesses an immunoreceptor tyrosine-based inhibition motif (ITIM) in its cytosolic tail, through which it recruits the inositol-5′ phosphatase SHIP, which regulates cell activation. Porcine FcγRIIb has been shown to mediate intrinsic antibody-dependent enhancement of PRRSV infection in PAM and transfected cell lines [72].

FcγRIIIa (CD16) was the first FcγR cloned and characterized in pig and the FcγR expressed at the highest level in PBMCs [13,14]. It is a 40 kDa type I transmembrane protein containing two extracellular Ig-like domains, followed by a transmembrane region and a 27 aa cytoplasmic tail [13]. Within the transmembrane region, there is a conserved eight aa sequence (LFAVDTGL), which in human, mouse and rat has been proposed to be involved in the interaction with FcRγ chain [73]. On the cell surface, CD16 is expressed as part of a multisubunit complex associated to FcRγ chain and other molecules that are required for proper cell surface expression and signal transduction [74]. Porcine CD16 is expressed predominantly on monocytes, macrophages, NK cells, neutrophils, and DC subsets [41,75]. It is expressed in all porcine monocytes, while in humans CD16 is only expressed on some monocyte subpopulations [76].

Splice variants, encoding different isoforms of porcine FcγRIIa [14], FcγRIIb [77,78] and FcγRIIIa [79], have been identified, but their functional significance remain to be determined.

### 2.4. CD163

CD163 belongs to a family of proteins, which in their structure contain scavenger receptor cysteine-rich (SRCR) domains. It is present in most tissue macrophages, and also in a subset of monocytes and, at lower levels, some blood DCs [19,41,80,81]. Porcine CD163 cDNA has been cloned and characterized, showing a high sequence homology with those of other mammal species [81]. It encodes a type 1 membrane protein, with an extracellular region consisting of nine SRCR domains, followed by a transmembrane segment and a short intracytoplasmic tail. Several variants of CD163 differing in their cytoplasmic tails as result of alternative splicing of the CD163 primary transcript have been reported [82].

In man, CD163 can bind hemoglobin–haptoglobin complexes, which are subsequently cleared from blood. Bound complexes are internalized and tissues are thereby protected from free Hb-mediated oxidative damage [83,84]. HMGB1-haptoglobin complexes also bind CD163, which results in the regulation of the inflammatory response in a heme-oxygenase 1 (HO-1)-dependent manner [85]. Both pro and anti-inflammatory mediators can modulate CD163 expression in monocytes and macrophages, which suggests a role of this molecule in the control of inflammatory processes [81,86,87,88,89]. 

CD163 can also act as a pattern recognition receptor binding either Gram-positive or negative bacteria, and triggering pro-inflammatory cytokine production [90]. On the other hand, it can be exploited by some pathogens to gain entrance into macrophages. Thus, CD163 has been involved in the entry and uncoating process of PRRSV infection [91]. Calvert et al. showed that transfection with CD163 cDNA of a variety of cell lines non-permissive to PRRSV infection rendered them fully permissive [82]. Requirement of CD163 for PRRSV infection has been more recently confirmed by in vivo experiments with CD163 knock-out pigs [92,93]. CD163 also appears to play a role in ASFV infection [94,95], although in this case it is not essential, as gene-edited pigs lacking CD163 were fully susceptible to infection by ASFV [96]. Nevertheless, anti-CD163 mAb 2A10 was found to inhibit binding of the ASF viral particle to PAM and interfere with virus infection [95]. More recently, Gao et al. observed that ASFV replicates in PK15 and 3D4-21 cell lines expressing both CD163 and Siglec-1, but not on the PK15 and 3D4-21 cells expressing only one of these receptors. Besides, simultaneous interference on CD163 and Siglec-1 expression in PAM with small interfering RNA (siRNA) significantly reduced the infectivity of ASFV [94]. These results point to a synergistic role of both receptors in the process of ASFV infection.

The extracellular region of CD163 can be cleaved by metalloproteases such as ADAM17 [97,98] and shed from the surface of monocytes and macrophages, in response to a variety of inflammatory stimuli. This soluble form of CD163 (sCD163) possesses immunomodulatory properties and a rise of its plasma levels is considered an indicator of an ongoing inflammatory or activation process of tissue macrophages [99,100]. Serum sCD163 can also bind the proinflammatory cytokine TWEAK, which reduces its biological activity [101]. Increased levels of sCD163 have been detected following infection with ASFV [102], PRRSV [103] or *Haemophilus parasuis* [104].

### 2.5. Siglecs

Sialic acid-binding immunoglobulin-like lectins (Siglecs) are so named by their property of binding sialic acid residues that usually are the terminal residues of glycans in glycoproteins and glycolipids. Siglecs are expressed on cells of the immune system and participate in processes that result in modulation of immune and inflammatory responses. All Siglecs have in their extracellular region a V-set Ig-like domain that contains the sialic acid binding site, and a variable number of C2-set Ig domains. Cytoplasmic tails of many Siglecs contain tyrosine motifs involved in signal transduction [105,106]. 

Siglecs have been classified into two groups: The first includes siglec-1 (also named CD169 or sialoadhesin) and siglecs-2, -4 and -15, and a second group includes siglec-3 (CD33) and CD33-related siglecs. Siglecs classified in the first group are not very homologous among them, but are conserved across evolution, and so, orthologues have been identified in all mammal species studied so far. Siglecs of the CD33-related group, on the contrary, are quite homologous in sequence of their extracellular segments and seem to be evolving rapidly; the number of genes coding for molecules including in this group is variable among species [107,108,109].

To date, only Siglec-1, -3, -5 and -10 have been characterized in swine [31,32,33,110,111], compared to 9 and 15 members of this family identified in mice and humans, respectively.

#### 2.5.1. CD169

CD169 also known as sialoadhesin (Sn) or Siglec-1, is the prototype of the Siglec family. From a structural point of view, CD169 shows the described structure of siglecs, containing 16 C2-set immunoglobulin domains, and a short cytosolic region. In swine, like in mouse and human, CD169 is expressed in various populations of tissue macrophages, but not in monocytes [112,113]. Yet, type I IFNs have been shown to be capable of inducing its expression in these cells [30,114,115]. In addition to an accessory function in the interactions of macrophages with other cells or the extracellular matrix [116], CD169 may play a role as a modulator of inflammatory and immune responses [105]. It can also contribute to the capture and phagocytosis of sialylated pathogens [117]. Furthermore, its high expression on macrophage populations located at antigen sites of entry and its function as a clathrin-dependent endocytic receptor have led researchers to evaluate its potential as an antigen-targeting receptor, using antigen immunoconjugates with Sn-specific ligands or mAbs to improve vaccination responses [30,118,119,120,121].

On the other hand, Sn has been shown to be involved in the entry of PRRS virus in macrophages, through the interaction of the α2-3 linked sialic acid residues present in the virion surface with Sn [110,122,123]. However, its role is not essential for PRRSV infection, as Sn-knock-out pigs show no increased resistance to infection [124]. 

Antibody binding to porcine CD169 causes a decrease in phagocytic capacity of PAM [125]. Likewise, the interaction of PRRSV with Sn also results in an impaired phagocytic capacity of macrophages [126]. Moreover, PRRSV appears to exploit its interaction with porcine Sn to inhibit the production of type I IFN through the DAP12 pathway and facilitate viral infection [127].

#### 2.5.2. Siglec-3/CD33 and CD33-Related Siglecs

Porcine Siglec-3 (CD33) and siglec-5 (CD170) are mainly expressed on cells of the myeloid lineage, whereas siglec-10 is predominantly found on B cells [31,32,33]. Precursors of the myeloid lineage express both Siglec-3 and siglec-5, although at very low levels; this expression increases during differentiation both in the monocytic and granulocytic lineages, reaching high levels of expression in mature monocytes and granulocytes, which has been related with the role of these molecules in the regulation of myeloid cell differentiation, and in the modulation of inflammatory response. In addition, these molecules are expressed in conventional DC and tissue macrophage populations; although, in these cells levels of expression are lower than in monocytes [31,32,128]. The presence of ITIM and ITIM-like motifs in the cytoplasmic segments of both receptors makes it possible that they share signaling pathways and therefore may have redundant functions or synergistic roles, activated upon recognition of distinct sialic acid-containing ligands; alternatively, they might regulate different processes [31,32].

Siglec-5 has been shown to participate in the regulation of innate responses to infection. In a human macrophage cell line, the overexpression of Siglec-5 upon transfection resulted in an inhibition of the production of TNF-α and an enhancement of that of IL-10 [129]. Several pathogens with sialic residues in their surfaces, like *Neisseria meningitidis*, group B *Streptococcus* (GBS) or human immunodeficiency virus type 1(HIV-1), have been shown to bind to human siglec-5 [117,130,131], interfering with the development of immune and inflammatory responses, which results in easier establishment of infection. The porcine pathogen *Glaesserella parasuis* encodes a sialidase, which interferes with the negative regulation of Siglec-5, mediated through the recruitment of SHP-2 tyrosine phosphatase, on the production of proinflammatory cytokines by PAM leading to an enhanced release of IL-1α, IL-6 and TNF-α and the consequent lung inflammation [132].

Siglec-10 has been proposed as an alternative receptor to Sn for PRRSV binding and entry, particularly for type 2 isolates. Although mainly expressed on B cells, it has also been detected on a minor subset of CD163^+^ spleen macrophages [111].

### 2.6. Beta 2-Integrins

Integrins are heterodimeric membrane proteins, consisting of an α subunit non-covalently bound to a β subunit, which are involved in cell-to-matrix and cell-to-cell interactions [133]. They are subdivided into different subfamilies based on their β subunits, which can associate with distinct α subunits. In humans and other species, the β2 subfamily, also named Leu-CAMs or leukocyte integrins, as they are exclusively expressed on leukocytes, comprises four members, each having a specific α subunit (CD11a, b, c and d) associated to the common β2 subunit or CD18 [134,135]. These molecules differ in their patterns of expression and have been shown to be involved in a variety of leukocyte adhesion-dependent phenomena, playing critical roles in inflammatory and immune responses [133,134]. CD11a is expressed on all leukocytes, whereas CD11b and c are preferentially expressed on myelomonocytic cells, and CD11d is restricted to tissue macrophages and some populations of lymphocytes [135]. 

While porcine CD11a shows a pattern of expression similar to that in other species, the potential porcine orthologues of CD11b and CD11c display a different cellular distribution compared to their human and murine counterparts [11]. No data have been reported for porcine CD11d.

At the Third International Swine CD Workshop, the cross-reactive anti-human CD11b mAb TMG 6-5 as well as the porcine-specific mAb MIL4 were shown to recognize in swine a heterodimer with the size expected for CD11b (165/95 kDa). However, the cellular distribution of this molecule differed from that of CD11b in human leukocytes, which is strongly expressed on all myelomonocytic cells, including granulocytes, monocytes, and macrophages, being detected in ≈70% of granulocytes but only marginally in a small proportion of monocytes (27–46%) and PAM (1.5–11.8%). This finding led to the provisional classification of these mAbs as wCD11R1 in the mentioned workshop [11]. Although porcine CD11b has been later cloned, no studies have addressed the reactivity of those mAbs with this molecule [136]. 

The cross-reactive anti-human CD11c mAb S-Hcl3, classified at the same workshop in wCD11R2, recognizes a heterodimer with an estimated size of 160/95 kDa [11]. Strikingly, whereas human CD11c is expressed on all myelomonocytic cells, this mAb shows moderate reactivity with monocytes, blood DCs and PAM, and do not label granulocytes [11,39]. However, recent cloning of porcine CD11c and generation of a specific mAb (3A8) against this molecule by Deloizy et al. showed that porcine CD11c is highly expressed on all granulocytes, monocytes, and blood DC1 and DC2 subsets, and at lower levels on T and NK cells [12]. These authors also confirmed the reactivity of anti-human CD11c mAb S-Hcl3 with porcine CD11c and speculated that the failure of this mAb to label porcine granulocytes may be due to alternative splicing or post translational modifications of pCD11c in these cells.

A third cluster, wCD11R3, defined at Third International Swine CD Workshop included several porcine-specific mAbs that precipitated a heterodimer with an estimated size of 155/95 kDa, strongly expressed on all myelomonocytic cells, including granulocytes, monocytes, and PAM [11], a pattern of staining similar to that of anti-CD11c mAb 3A8 [12]. Although two of these mAbs (2F4/11 and C25) were originally reported as recognizing a β2-integrin, involved in the phagocytosis of complement-opsonized zymosan particles and in recognition of β-glucans by macrophages, that might correspond to porcine CD11b [137,138,139], similarity with 3A8 reactivity suggests that they may recognize porcine CD11c. 

### 2.7. Toll-Like Receptors (TLRs)

Among pattern recognition receptors (PRRs), Toll-like receptors (TLRs) are one of the most ancient families. In the pig, genes coding of 10 different TLRs have been cloned and the encoded products (TLR1-TLR10) characterized [140,141]. Structurally, TLRs are type I transmembrane proteins, and the extracellular domain is a structure composed of leucine-rich repeats, involved in ligand binding and microbial sensing. The cytoplasmic domain, named the TIR domain for its homology with that of interleukin 1 (IL-1) receptor, interacts with MyD88 (common to all TLRs except TLR3) and TRIF (only used by TLR3 and TLR4) adaptors for signaling through two different pathways [142]. Signaling through TLRs induces expression of co-stimulatory molecules and production of type I interferons (IFNs), chemokines and pro-inflammatory cytokines [143,144]. Different TLRs induce distinct but overlapping patterns of expression of inflammatory genes; thus, integration of the simultaneous delivery of signals caused by the binding of various TLRs by different microbial components might contribute to shaping the response to the invading pathogen [145,146].

Usually, the main form of TLRs in the membrane is a dimer, some homodimers and other heterodimers (e.g., TLR2/TLR1 and TLR2/TLR6). Localization of some of these receptors (TLR1, TLR2, TLR4, TLR5, TLR6 and TLR10) is mainly at the cell surface, and these largely recognize microbial surface components, such as proteins, lipoproteins, or lipids, whereas others (TLR3, TLR7, TLR8 and TLR9) are mainly expressed within intracellular compartments and recognize nucleic acids. Each TLR detects distinct structural components of pathogens. Thus, TLR2 may recognize different PAMPs present in Gram-positive bacteria, but also viral proteins [147]. Dimers composed of TLR2 and TLR6 are involved in responses to diacylated lipoproteins, while dimers of TLR2 and TLR1 are necessary for recognition of tri-acylated lipoproteins. TLR3 interacts with virus-derived double stranded RNA, while the main ligand of TLR4 is the LPS of Gram-negative bacteria, but it also detects fusion and envelope proteins from viruses. Flagellin, the main component of the bacterial flagella, binds TLR5. TLR7 and TLR8 recognize viral single-stranded RNA; and TLR9 senses unmethylated CpG DNA derived from bacteria, viruses, or parasites. TLR10, when dimerized with TLR2, recognizes ligands from *Listeria* sp but can also sense influenza A virus infection [143]. Endogenous ligands can also be recognized by some TLRs, so TLR4 interacts with heat shock proteins, fibronectin fragments or fibrinogen, which might alert of stressful or danger conditions. 

Using monoclonal and/or polyclonal antibodies in flow cytometric, immunohistochemical and Western blot analyses, expression of TLR2, TLR3, TLR4, TLR6, TLR7 and TLR9 has been reported on porcine PAM and other macrophages populations [34,35,36,37,148,149,150,151,152].

### 2.8. C-Type Lectin-Like Receptors

Myeloid C-type lectin-like receptors (CLRs) are a group of PRRs that recognize microbial components as well as endogenous ligands released from stressed or damaged cells, playing important roles in host defense against pathogens and in the maintenance of homeostasis [153]. Many of these receptors signal to modulate myeloid cell activation and thereby regulate innate and adaptive immune responses. In addition, some can act as endocytic receptors that mediate the uptake of antigens for presentation to T cells [153,154,155].

CLRs have been classified into several groups (I-XVII) according the arrangement of their C-type lectin-like domains (CTLDs) and phylogenetic considerations, but also depending on the signaling motifs present in their cytoplasmic tails [153,156]. Some CLRs may transmit signals either by ITAM-like motifs in their cytoplasmic tails (e.g., Dectin-1), or through the motifs in adaptor molecules they associate with (e.g., Dectin-2). Signaling after activation of these receptors occurs through spleen tyrosine kinase (Syk)-dependent pathways, which induces nuclear factor-kB (NF-kB)-dependent pro-inflammatory responses [157,158]. Other group of CLRs, like CLEC-12A, have ITIM motifs in their cytoplasmic portions, and activation upon receptor engagement promotes recruitment of tyrosine phosphatases like SHP-1 or SHP-2, which in turn regulate negatively kinase-associated pathways that may had been activated by heterologous receptors, resulting in the inhibition of cellular activation [159]. In addition, features of the ligand binding process may lead to distinct signals through the same motif, by triggering alternative pathways. CLR ligands are frequently part of complex structures that can simultaneously bind different receptors, resulting in different outcomes depending on the crosstalk among these receptors [160].

In swine, CLRs are still being identified, and only a few have been characterized, in contrast with the many members of this family identified in mice and humans. Out of them, CD206, CD209, dectin-1, CLEC12A and CLEC12B are expressed on macrophages [27,28,161,162].

#### 2.8.1. CD205

CD205, or DEC-205, is an endocytic receptor. It has been extensively studied mostly in mice and its characteristics make it a suitable target receptor for the delivery of antigens and modulation of immune response through a more efficient processing and presentation by MHC molecules [153,163]. It is a type-I transmembrane protein, its extracellular portion is composed of a ricin-type beta-trefoil (RICIN) domain, a fibronectin type-II (FNII) domain, and ten C-type lectin-like domains (CTLDs), and it has a short (31 aa) cytoplasmic domain [164]. 

Porcine CD205 shows a broad distribution, being expressed on DC subsets of blood, skin, tonsil, spleen, and mesenteric and submaxillary lymph nodes as well as on monocytes [24,165,166]. However, there are no studies on the expression of this receptor on tissue macrophages, although in humans it has been detected on these cells [167].

#### 2.8.2. CD206/Mannose Receptor 

The mannose receptor (MR, CD206) is a type I transmembrane protein with an extracellular region comprising an N-terminal cysteine-rich domain, a fibronectin type II domain, and eight CTLDs, followed by a transmembrane segment and a short cytoplasmic domain. It binds to glycan structures bearing mannose, fucose, and N-acetyl glucosamine residues, which are present on the surface of many microorganisms (viruses, bacteria, fungi, and protozoa) and self-molecules, contributing to their endocytosis via clathrin-coated vesicles [168]. The uptake of antigens by the MR allows for processing and presentation via both the MHC class I and II pathways, which makes this receptor a suitable target for antigen delivery for vaccine development [155]. Porcine MR is expressed by macrophages and DCs [89,169,170]

#### 2.8.3. CD209/DC-SIGN

Dendritic cell-specific intercellular adhesion molecule-3-grabbing nonintegrin (DC-SIGN), also known as CD209 or Clec4L, is a type II transmembrane CLR. It has an N-terminal short cytoplasmic tail, in which a dileucine-based internalization motif has been identified; after the transmembrane region, there is a flexible domain, usually referred to as neck, which has been shown to be involved in oligomerization, and finally a Ca ^2+^-dependent CTLD. DC-SIGN binds carbohydrates present on pathogens that contain mannose, fucose, N-acetyl galactosamine, and N-acetyl glucosamine residues, and mediates their endocytosis, which results in activation and shaping of the adaptive immune response against them. The porcine homolog of DC-SIGN has been cloned and characterized by Huang et al., and found to be expressed on monocyte-derived macrophages and dendritic cells, PAM, and macrophage- and dendritic-like cells in lymph node sinuses. It was also detected on lymph node endothelial cells, but not in liver cells or monocytes [161].

#### 2.8.4. Dectin-1/CD369

Dectin-1, also known as CLEC7A or CD369, recognizes structures of Mycobacteria, plant cell walls, fungi, and yeast, which contain β-1,3-linked glucans. It has been determined that it is essential in processes leading to protective immune response to *Candida albicans* and other fungi in mice and humans [171,172]. In pigs, dectin-1 has been shown to be involved in the phagocytosis of zymosan by macrophages [139]. Porcine dectin-1 has been cloned by Sonck et al., which identified transcripts for three different major isoforms of this molecule in PAM [162]. The larger isoform (isoform A) consists of a cytoplasmic region on the N-terminal end, which contains a hemi-immunoreceptor tyrosine-based activation motif (hemITAM), a transmembrane region, a stalk region and a single C-terminal CTLD. The second major isoform (isoform B) lacks the stalk region, resulting in a truncated protein, whereas the third isoform is a variation of the primary isoform with a deletion in the transmembrane and stalk region.

#### 2.8.5. CLEC12A/CD371

CLEC12A, also known as MICL or CD371, is a type II transmembrane protein classified within group V of CLRs. We have recently characterized the porcine homolog of CLEC12A [27] Its extracellular portion contains only one CTLD domain connected, via a stalk region, to the transmembrane segment. The cytoplasmic N-terminal segment of this protein has only 42 aa and contains an ITIM motif. It is usually expressed as a dimer (30 kDa/60 kDa) on porcine PAM. Regarding its pattern of expression, it seems to be more restricted than that of mouse or human homologs [173,174], being only expressed in blood plasmacytoid and conventional DCs (cDC), with a higher expression on cDC1 relative to cDC2, and PAM, but not on monocytes or granulocytes.

#### 2.8.6. CLEC12B

CLEC12B, also named as macrophage antigen H (MAH), is included in the Dectin-1 cluster, classified within group V of CLRs [175]. The structure of the porcine homolog of CLEC12B resembles that of CLEC12A described above. Porcine CLEC12B is expressed on PAM and, at lower levels, on blood cDC1 and plasmacytoid DCs. No expression could be detected on other blood cells [28].

### 2.9. CD107a/Lamp1

CD107a, also known as the lysosome-associated membrane protein-1 (lamp-1), is one of the major glycoproteins on the membrane of lysosomes, but can also be detected in small amounts on the surface of macrophages, activated platelets and other cell types, such as cytotoxic T lymphocytes (CTL), NK cells and granulocytes [176,177,178]. In CTL and NK cells, CD107a has been used as a marker of activation, because its abundant expression in the membrane of cytotoxic granules becomes incorporated into the plasma membrane of these cells during lethal hit delivery [179,180]. 

Structurally, CD107a is a type I membrane glycoprotein, with a large, heavily glycosylated domain located on the luminal side of lysosomes, anchored by a transmembrane region with a short cytoplasmic tail [181]. The intralumenal domain consists of two related subdomains of approximately 160 residues separated by a hinge region rich in proline residues [182]. This domain contains a high number of potential N-glycosylation sites, which makes this molecule one of the most densely N-glycosylated proteins characterized so far, with carbohydrates accounting for 55–65% of its total mass [176]. 

MAb 4E9/11 recognizes porcine CD107a/Lamp-1. This antibody strongly stains tingible body macrophages within the follicles of secondary lymphoid organs and can be used in immunohistological analysis of formalin-fixed, paraffin-embedded tissues [183]. In the spleen, this antibody labels macrophages of red pulp and ellipsoids, and a minor population within the T cell area of periarteriolar lymphoid sheath. It also labels macrophages scattered in T cell areas and within the sinuses of lymph nodes, in the cortex and medulla of thymus, in the *lamina propria* of gut, and the Kupffer cells of liver [17].

### 2.10. CD68

Another member of the lysosome-associated membrane protein family is CD68, which has been frequently used as a pan-macrophage marker in analyses of mouse and human tissues. Anti-human CD68 mAb EBM11 has been shown to cross-react with pig cells in the animal homologue section analyses of the Eighth Human Leucocyte Differentiation Antigen (HLDA8) Workshop [16].

### 2.11. CD115/CSF1R

CD115, or CSF-1R, is a member of the family of type III growth factor receptors with tyrosine kinase activity, which controls the survival, proliferation, and differentiation of cells of the mononuclear phagocyte system [184,185]. It binds to CSF-1, also named M-CSF, and IL-34. In mice and humans, a high level of expression of CSF1R mRNA has been shown to be restricted to macrophages and their progenitors [186].

The porcine homolog of CSF-1R has been cloned by Hume and colleagues, who also developed several mAbs (ROS8G11, ROS3A5 and ROS3B10) against this molecule [18,187]. The extracellular region of the molecule consists of five Ig-like domains (one V-set and four C2-set). The cytoplasmic region contains a protein tyrosine kinase domain and ATP-binding region required for the catalytic activity of the receptor upon the binding of CSF-1 or IL-34 to the extracellular domain. MAbs against this molecule stain PAM, monocytes as well as bone marrow progenitors that have been differentiated with recombinant human CSF-1. Mouse and human CSF-1 and IL-34 can bind and activate the porcine CSF-1R [187]. 

### 2.12. CD200R Family

CD200R1 and CD200R1L constitute a family of paired receptors—that is, highly homologous proteins that, due to significant differences in their cytoplasmic segments, are capable of transmitting opposing types of signals. They are transmembrane type I glycoproteins with two almost identical Ig-like domains in their extracellular portion [188]. The CD200R1 cytoplasmic segment contains three tyrosine residues, leading to the inhibition of the ERK signaling pathway by recruiting the adaptor molecule Dok2 and subsequent activation of RasGAP [189,190,191]. On the other hand, a lysine residue in the transmembrane segment of CD200R1L mediates association with DAP12, a signaling protein whose ITAM motif in its cytoplasmic tail allows for the recruitment and activation of tyrosine kinases leading to cell activation [188,192].

CD200R1 interacts with CD200, a glycoprotein expressed on the surface of a broad variety of cells, through their respective N-terminal Ig-like domains. However, there is no consensus about the binding of CD200R1L molecules to CD200, despite the high aa sequence homology of its extracellular region with that of CD200R1 [188,193,194]. 

Porcine CD200R1 and CD200R1L are expressed on PAM, monocytes, and B cell subsets but not in T or NK cells, unlike their murine and human homologues, which are also expressed on the latter cells [22,188,195]. Alternative splicing variants of these receptors have been detected in porcine PAM and monocytes, but their biological functions remain to be determined [22,196]

In humans and mice, CD200R1 and CD200R1L have been implicated in the regulation of the production of pro-inflammatory mediators in macrophages [197,198,199,200]. In addition, the expression of CD200R1 has been associated with alternative macrophage polarization. In humans, CD200R1 expression can be induced in monocyte-derived macrophages with the addition of IL-4 or IL-13, which leads to M2a macrophages that exert anti-inflammatory functions [201,202]. However, in mouse, IL-4 and IL-13 fail to induce CD200R1 expression, but this is down-regulated by treatment with IFN-ɣ [201]. Similarly, porcine monocytes cultured with IFN-ɣ down-regulate the expression of CD200R1, whereas this is increased in the presence of IL-10 but not of IL-4 or IL-13 [196].

### 2.13. CD203a

Porcine CD203a, originally clustered as SWC9 at the Second International Swine CD Workshop [203], is the homolog of human ecto-nucleotidepyrophosphatase/phosphodiesterase 1 or ENPP1, a type II transmembrane glycoprotein highly expressed on lung macrophages and other macrophage populations but not on monocytes [23,204]. It is up-regulated during the differentiation of these cells into macrophages, having been used as a marker for discrimination between both cell types [19,205,206]. However, its expression is not restricted to these cells, as it is also present on thymocytes [203]; high levels of transcripts have also been reported in muscle [207].

CD203a cleaves ATP and 2′3′ cyclic GMP-AMP (cGAMP) to AMP and GMP, contributing to the control of the inflammatory responses, as extracellular ATP is sensed by purinergic receptors, and mediates NLRP3-inflammasome activation, while cGAMP activates STING, which can initiate an inflammatory response by using the TANK-binding kinase 1 (TBK1)–IRF3 pathway, resulting in the production of type 1 interferons (IFNs) and other cytokines [208,209,210]. A role of CD203a (ENPP1) in the replication of pseudorabies virus (PRV) has been proposed, by regulating cGAMP homeostasis and thus inhibiting IRF3 activation and IFN-β production [207]. 

### 2.14. F4/80 or ADGRE1

The F4/80 antigen, encoded by the *Adgre1* locus, has been used to identify mouse macrophage populations in a wide range of tissues [211]. It is a multispanning transmembrane G protein-coupled receptor in whose extracellular region are found repeated Epidermal Growth Factor-like calcium binding domains. Waddell et al. have developed a mAb named ROS-4E12-3E6 against the porcine homologue of F4/80 antigen or ADGRE1 [29]. Using this mAb, porcine ADGRE1 has been shown to be highly expressed in tissue macrophages, monocytes, and mature granulocytes. Comparative analyses among mammalian species reflect a rapid evolution of this molecule, which is consistent with a role in pathogen recognition.

## 3. Origin and Plasticity of Tissue-Resident Macrophages

In steady state conditions, resident tissue macrophages exhibit a marked heterogeneity in morphology and expression of cell surface antigens with unique phenotypes in distinct microenvironments. This heterogeneity, which is further increased by activation, is a reflection of their ontogeny and functional specialization within different tissue niches and is most likely regulated by distinct master transcription factors, which are expressed in a tissue-specific and niche-specific manner [212,213,214].

Until recently, monocytes were considered the sole precursors of tissue macrophages in adult life. However, in the last two decades, fate-mapping studies in mice have shown that, in many tissues, macrophage populations are derived from yolk sac or fetal liver progenitors that populated those organs during embryonic development and are maintained in the steady state by self-renewal, while circulating monocytes contribute to the reinforcement of macrophage populations during inflammation [215,216,217].

In pigs, knowledge about the ontogeny of macrophages is scarce. Rehakova et al. described a prominent population of CD45^+^ CD172a^+^ cells in yolk sac on the 21st day of gestation (DG21, full gestation 114 days), although no phagocytic activity was evidenced. Moreover, at DG21, cells with a similar phenotype (CD45^+^ CD172a^+^ cells) could be isolated from fetal liver and their frequency gradually increased with fetal age. On day DG25, cells with macrophage morphology ingesting apoptotic bodies were detected in fetal liver [218]. In a different study, Bordet et al. showed that porcine PAM and pulmonary intravascular macrophages (PIM), like murine PAM, display a gene expression profile typical of macrophages from embryonic origin rather than those derived from adult bone marrow monocytes, with a strong expression of the HDAC10 and PU.1 markers but low levels of the hematopoietic cell marker c-Kit [219].

### 3.1. Macrophage Polarization

The phenotypic heterogeneity displayed by tissue macrophage populations can be further increased by the variety of activation states that these cells may adopt in response to distinct endogenous or exogenous stimuli found in their local environment, such as cytokines, microbes, microbial products and other modulators like nucleotide derivatives or glucocorticoids [220]. In this regard, macrophage activation should be envisaged as a dynamic process in which these cells keep the capacity to reprogram their phenotype and function to respond to new changes in surrounding conditions, such as those that take place along the different phases of an inflammatory response [221,222,223]

In mice and humans, activated macrophages have been classified into two major groups: classically activated macrophages (M1 Mφ) and alternatively activated macrophages (M2 Mφ). The latter, in turn, are subdivided into M2a, M2b, M2c and M2d based on the activation stimuli and gene expression profiles [224,225,226,227]. However, this classification represents a simplified view that does not account for the broad spectrum of activation states that may result from the complex in vivo environments of many macrophages, in which multiple cytokines and soluble factors interact to imprint their functional state, and which explains why many surface markers identified on in vitro generated macrophages fail to translate to macrophages in vivo, [214,228], which has led to the proposal of naming activated macrophages by denoting the cytokines, growth factors and other signals that caused a specific macrophage state [220]. Nevertheless, this M1/M2 classification is still frequently used, as it provides a useful framework for approaching the analysis of macrophage activation in different pathologic processes. 

Activation of NF-kB and STAT1 signaling pathways by IFN-γ alone or together with LPS, or other TLR agonists, or also with TNF-α or GM-CSF, induces M1 polarization of Mφ. These M1 Mφ produce high levels of IL- 12 and other pro-inflammatory cytokines (IL-1β, IL-6, TNFα), and exhibit potent microbicidal capacities, via the production of reactive oxygen intermediates and nitric oxide (NO). They also have enhanced antigen-presenting functions promoting strong Th1-polarized immune responses [229]. 

M2a Mφ are induced by IL-4 or IL-13, which activate STAT6, and characterized by high expression of CD206, the decoy receptor IL-1 receptor 2 (IL-1R2) and arginase 1 (Arg-1) and the secretion of cytokines that contribute to tuning down inflammation, repairing damage and regaining tissue homeostasis, such as TGF-β and IL-10. M2b Mφ are induced by immunocomplexes, TLR ligands or IL-1β and secret pro-inflammatory and anti-inflammatory cytokines such as TNF-α, IL-1β, IL-6, IL-10, and CCL1, and are particularly effective as APC, in contrast to other M2 Mφ, promoting the development of T cells that produce high levels of IL-4 and IL-10 [230]. M2c Mφ can be generated with IL-10, TGF-β or glucocorticoids; they express CD163 and CD206, and produce IL-10 and TGF-β, displaying anti-inflammatory characteristics. M2d Mφ are induced by adenosine receptor ligands and TLR ligands and are characterized by their ability to promote angiogenesis and tumor progression by means of IL-10 and VEGF production [231].

Gene expression analyses have allowed for the identification of sets of specific markers for different subtypes of activated macrophages in mice and humans [232,233]. These analyses reveal differences among species, for instance, whereas arginase-1 expression is one of the discriminative criteria between classically and alternatively activated murine macrophages, IL-4 does not induce its expression in human monocyte-derived macrophages [234]. Likewise, human macrophages do not produce NO in response to LPS [235,236]. These differences probably reflect different evolutionary outcomes sculpted by environmental factors [220].

**Figure 1:**
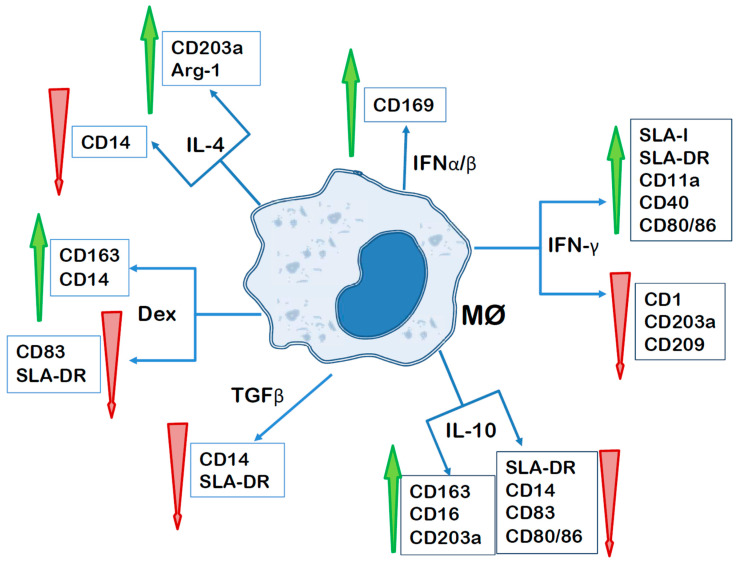
Phenotypical changes in monocyte-derived macrophages following treatment with cytokines or glucocorticoids (Dex: dexamethasone). Based on [89,115,237,238].

Several groups have addressed the changes in phenotype and function of porcine macrophages in response to specific stimuli or conditions (Figure 1). Singleton and colleagues compared the phenotype of monocyte-derived macrophages that have been treated for 24 h with LPS and recombinant porcine IFN-γ for classical activation (M1 Mφ), or with either recombinant porcine IL-4, recombinant porcine IL-10 or dexamethasone for alternative activation (M2 Mφ) [89]. M1 Mφ expressed higher levels of SLA-DR and CD80/86, and lower levels of CD209 than unstimulated and M2 Mφ. On the other hand, IL-4 and IL-10 induced the up-regulation of CD203a, but not of CD206, whereas treatment with dexamethasone or IL-10 significantly up-regulated CD163 expression and decreased that of CD83. IL-10 also decreased CD80/86 expression. 

Sautter et al. also reported a significant increase in the expression of molecules involved in antigen presentation, such as MHC-I and MHC-II, and others like CD11a and CD40, in monocyte-derived macrophages treated with IFN-γ, which was accompanied by a down-modulation of CD1 and CD203a [115]. These authors also observed an up-regulation of CD203a after treatment of porcine monocyte-derived macrophages with IL-4, but not of the mannose receptor CD206, in contrast to what has been described with murine and human monocyte-derived macrophages [202,239,240]. CD169 expression was found to be upregulated by type I IFNs, in agreement with previously seen with porcine IFN-α in monocytes and macrophages [30,114]. Porcine M1- and M2-polarized macrophages also differed in the pattern of cytokines they produced. Porcine IFN-γ-primed monocyte-derived macrophages (M1) secreted higher amounts of IL-12p40 and IL-6 in response to LPS stimulation, compared to IL-4-treated monocyte-derived macrophages (M2), whereas no differences were observed in the production of IL-10, TNF-α, IL-1β or IL-8 between these two populations [115]. These results were interpreted as in the pig. IFN-γ promotes the antigen-presenting capacities of macrophages and favorable conditions for the development of Th1 cell responses, without a clear pro-inflammatory and antimicrobial status. On the other hand, IL-4 induces the expression of Arg-1 and CD203a, without clearly impacting pro-inflammatory responses.

Like human macrophages, and in contrast to murine macrophages, porcine macrophages do not produce NO in response to LPS [241,242]. 

Carta, Franzoni and colleagues have assessed more recently the changes that happened in monocyte-derived macrophages after treatment with either classical activation stimuli or various M2-polarizing factors [237,238]. Classical activation (IFN-γ + LPS) induced higher expression of MHC molecules, in line with previous studies [115]. On the contrary, M2-polarizing factors, IL-10, TGF-β or dexamethasone, down-regulated the expression of MHC class II molecules. IL-10, TGF-β, as well as IL4, also reduced the expression of CD14, which was up-regulated by dexamethasone, in contrast with what happened in human cell lines [243]. Expression of CD163 was up-regulated by dexamethasone and IL-10, but not TGF-β, which agrees with data reported previously by Singleton et al. [89]. CD16 expression was also enhanced by IL-10.

Regarding cytokine production, in these studies, treatment with IFN-γ+ LPS resulted in an increased production of a higher number of pro-inflammatory cytokines (IL-1α, IL-1β, IL-6, IL-12, IL-18, TNF-α and CXCL8), compared with that observed in Sautters’ study [115]. IFN-γ + LPS stimulation also resulted in the release of significant levels of IL-1 receptor antagonist (IL-1Ra) and small amounts of IL-10, which can be envisaged as a protective mechanism to prevent detrimental inflammatory responses. Production of proinflammatory cytokines in response to TLR2 or TLR4 agonists was abolished by treatment with IL-10 or dexamethasone, whereas treatment with TGF-β was much less effective. Arg-1 expression could not be triggered by either IL-10 or TGF-β, whereas a marked induction was observed after IL-4 treatment, in agreement with the results previously reported by Sautter et al. [115]. Moreover, no release of IL-10 was observed in porcine macrophages exposed to IL-4 (M2a), or IL-10, TGF-β or glucocorticoids (M2c) [237,238], unlike human or murine macrophages that produce high amounts of IL-10 in response to these factors [229,244,245,246].

### 3.2. Macrophage Populations in Different Organs

As previously mentioned, tissue macrophages exhibit a marked heterogeneity, which is further increased by activation; this heterogeneity is quite evident when comparing macrophages residing in different tissues. In pigs, macrophage populations have been characterized in several organs, such as spleen, lymph nodes, tonsil, lung, liver, placenta, and skin. In this section, we will review current knowledge on macrophage populations in those organs (Table 2).

#### 3.2.1. Spleen

Analysis of the expression of CD163 and CD169 identifies two major macrophage populations in porcine spleen: CD163^+^ CD169^−/lo^ and CD163^−^ CD169^+^. CD163^−^ CD169^+^ macrophages are located in the marginal zone and ellipsoids, while CD163^+^ CD169^−/lo^ macrophages are mainly found in the red pulp [30,81,121,152]. These two subsets also differ in the expression of CD11b, which was detected at high levels in the majority of spleen CD163^−^ CD169^+^ macrophages while CD163^+^ CD169^−/lo^ cells were CD11b^−/lo^. CD169^+^ macrophages also express higher levels of SLA-DR and costimulatory molecules (CD80/86) than CD163^+^ macrophages. However, the latter display a higher ability to present soluble antigens to CD4^+^ T cells in a secondary in vitro response [152].

Another population of tingible body macrophages, involved in the uptake and digestion of apoptotic B lymphocytes can be identified within the follicles in the white pulp by the high expression of Lamp-1/CD107a [17].

In mouse, two phenotypically and functionally different subsets of macrophages had been described in marginal zone: metallophilic macrophages (CD68^+^ CD169^+^) involved in virus clearance, and marginal zone macrophages (CD68^+^ MARCO^+^ SIGN-R1^+^), involved in tolerance against blood-borne apoptotic cells [4,258]. Whether these subsets are also present in swine remains to be determined. 

#### 3.2.2. Lymph Nodes

Compared to mouse and human, swine lymph nodes (LN) have an inverted structure, which is also present in elephants, dolphins, and hippopotamus, with the lymph flowing from the center to the periphery [248,259,260]. 

The first studies of porcine LN with antibodies to CD163 and CD169 identified a major population of macrophages that co-expressed both markers located in the peripheral region of LN. In addition, a population of CD163^−^ CD169^+^ cells was detected on the rim of B cell follicles and inside them [30,121]. The CD163^−^ CD169^+^ population contained some cells that expressed high levels of CD11b, and were able to present soluble antigens to T cells in vitro [152]. Since CD11b expression in porcine lymphoid tissues had been previously associated to DC [261], it was speculated that these cells might be related to murine CD169^+^ DC described by Berney et al., which have the capacity to attract and provide antigenic stimulation to lymphocytes [262]. More recently, Bertho and colleagues have carried out an elegant and detailed analysis of the structure of porcine LN and its different macrophage populations [247,248]. They characterized three macrophage populations: (i) the CD163^−^ CD169^+^ macrophages found at the periphery of the B cell follicles and thought to be the counterpart of the murine subcapsular sinus macrophages, which are involved in the translocation of soluble antigens from the afferent lymphatic sinus to the inside of the follicle; (ii) the CD163^+^ CD169^+^ macrophages found in the periphery of LN, before the efferent lymphatic vessels, are considered the functional equivalent of the mouse medullary sinus macrophages, which would be involved in the clearance of the lymphatic fluid before its exit to the main blood circulation; (iii) the CD163^+^ CD169^−^ macrophages positioned along the medullary cords with a phenotype similar to the murine medullary cord macrophages (MCM), which have a role in the plasma cell terminal maturation [263,264].

#### 3.2.3. Tonsil

In pigs, the palatine tonsil is positioned at the opening of the respiratory and gastrointestinal tract, providing a first line of defense against air-borne pathogens. Soldevila and colleagues addressed the characterization of myeloid cell populations residing in this organ identifying five distinct populations comprising macrophages, a putative CD14^+^ moDC population, two subsets of conventional DCs (cDC1s and cDC2s) and plasmacytoid DCs [249]. Tonsil macrophages were identified as MHC-II^hi^ CD172a^hi^ CD4^−^ CADM1^lo^ CD14^−^ (using the cross-reactive mAb Tük4) CD163^+^. These cells express abundant CSF1R, MAFB, IL-1B, TLR2, TLR4, and TLR6 transcripts and display a high capacity to capture and process antigen but a low capacity for naïve T cell stimulation. They, as well as the CD14^+^ population, were found located close to areas where pathogens might be expected to enter the tonsil, being abundant within the crypts, in the epithelium, the subepithelial connective tissue and the adjacent lymphoid tissue [249,265]. 

#### 3.2.4. Liver

Kupffer cells, the resident macrophages of the liver, are located in the hepatic sinusoids, where they are exposed to the constant flow of blood. They serve as a first line of defense, removing foreign debris, particles, and potential pathogens passing from the gastrointestinal tract via the portal circulation. They are also involved in the clearance of apoptotic cells and senescent erythrocytes from the systemic circulation, being responsible for recycling hemoglobin [266]. Porcine Kupffer cells are positive for CD107a, CD163 and CD169 [17], and they have been extensively studied in the context of liver xenotransplantation, where they play a major role in sequestration of human platelets and red blood cells. Porcine Kupffer cells phagocytose human platelets through β2-integrins and CD40 [267,268]. They also recognize human erythrocytes by a sialic acid-dependent mechanism involving CD169 [269,270]. 

#### 3.2.5. Lung

PAM can be easily obtained from bronchoalveolar lavages and have been used extensively in the characterization of porcine macrophage receptors and to study the interaction of these cells with relevant swine pathogens such as ASFV, PRRSV or *Actinobacillus pleuropneumoniae* [271,272,273]. PAM express high levels of CD163, CD169, CD172a, CD203a and CD206/MR, and low levels of CD14 and CD16 [30,63,206,219,250,274].

Studies with animals that have been depleted of alveolar macrophages by treatment with liposome-encapsulated dichloromethylene diphosphonate point to an essential role of these cells in protection against influenza virus infection in pigs [251].

In addition, lung parenchyma contains a population of intravascular macrophages with phenotypic characteristics similar to those of PAM. These PIM, which are also present in cattle, sheep, goat, cats, horses, and cetaceans, possess strong phagocytic and bactericidal capacities, and like Kupffer cells in the liver, are involved in the clearance of blood-borne particles and pathogens [253,275]. They also support replication of PRRS, ASF or CSF viruses [219,252,276,277]. Both PAM and PIM display a gene expression profile characteristic of embryonic-derived macrophages, with a strong expression of the macrophage-associated gene MerTK, HDAC10 and PU.1, and low expression of genes specific to hematopoietic cells such as c-Kit, CCR2 or CX3CR1, [219,274]. Lung parenchyma also contains a CD172a^int^/CD163^int^ population of parenchymal cells that present monocyte-derived cell characteristics but also display macrophage features. These cells, named as moMacro, are CD11b-like^+^ CD1^−^ CadM1^lo^ MR-like^+^ and CD14^+^ [170,274].

#### 3.2.6. Nasal Mucosa

Nauwynck’s group characterized macrophage populations in nasal mucosa, as this represents a primary entry site for many pathogens, including PRRSV [254,255]. These authors identified two major populations: CD163^+^ CD169^−^ cells, located in the upper *lamina propria* and within the epithelium, and CD163^+^ CD169^+^ cells, predominantly found in the deeper area of *lamina propria*, close to the cartilage. A minor population of CD163^−^ CD169^+^ cells was also detected in the submucosa close to the cartilage. The CD163^+^ CD169^−^ macrophages display a different susceptibility to high and low virulent PRRSV strains and may be useful in studies aiming to identify alternative receptors to CD169 for binding and internalization of PRRSV in macrophages [255]. 

#### 3.2.7. Skin

Pig skin contains a population of dermal macrophages with a phenotype CD172a^+^ CD163^+^ SLA-DR^−/lo^, CD14^+^, CD16^+^, DC-SIGN/CD209^lo^, MR/CD206^lo^, CD1^−/lo^ [25,169]. A phenotypically similar population, MHC-II^lo^ CD163^hi^, has been described in human dermis [278]. CD163 is also expressed on two populations of dermal dendritic cells (DDCs): one CD163^hi^, with CD163 levels similar to those of macrophages, and other CD163^lo^. The CD163^hi^ DDCs are, like macrophages, CD14^+^ CD16^+^ DC-SIGN/CD209^lo^ MR/CD206^lo^, but express higher levels of SLA-DR and CD1a. Although morphologically different from dermal macrophages, these cells express as much CSF1R/CD115, MAFB, and CD64 transcripts as dermal macrophages, and, according gene expression analyses, appear to be the equivalent of the monocyte-derived human CD14^+^ DDCs [169]. Regarding the CD163^lo^ DDCs, they are negative for DC-SIGN and CD206, expressed lower levels of CD14 and CD16, and higher levels of SLA-DR and CD1a. 

#### 3.2.8. Placenta

Karniychuk and Nauwynck investigated changes in porcine macrophages of sow and fetus organs along gestation, as these cells represent potential targets for PRRSV replication [256,279]. In the endometrium and fetal placentas, two major macrophage subsets (CD163^+^ CD169^−^ and CD163^+^ CD169^+^) were observed. At all gestational stages, high numbers of CD163^+^ CD169^+^ macrophages were found in the endometrium, scattered within connective tissues and close to blood vessels. The highest numbers of CD163^+^ macrophages in fetal placentas were observed at late times of gestation. At 114 days of gestation, macrophages were lined along the fetal trophoblast, and many of them were located close to fetal blood vessels. CD169^+^ macrophages were present in fetal placentas, scattered all over the connective tissues, at all gestational states except during mid-gestation (days 50–60), which might explain the difficulty for PRRSV to spread transplacentally at this stage of gestation.

Novakovic et al. also analyzed macrophage populations of endometrium and fetal placenta in pregnant gilts infected with a type 2 PRRSV isolate. The number of CD163^+^ macrophages in the endometrium as well as the fetal placenta exceeded that of CD169^+^ macrophages, and both were increased in infected animals. In their study, the largest number of CD163^+^ macrophages was found to reside in the fetal placenta near maternal and fetal microvilli interdigitation. A positive correlation was observed between CD163^+^ cell counts in endometrium and PRRSV load in fetal thymus [257]. 

## 4. Porcine Macrophage Cell Lines

Among various populations of porcine macrophages, PAM and monocyte-derived macrophages are the most frequently used in research studies on innate immune functions and host–pathogen interactions, because of their easy obtention. However, use of these primary cultures has some drawbacks, besides their cost, as they require a regular supply from donor animals, exhibit significant variability among batches and animals with different genetic backgrounds, and entail ethical concerns associated with the use of animal tissues. These drawbacks have spurred numerous efforts to develop immortalized porcine cell lines that display and maintain functional characteristics of primary macrophages as close as possible. These immortalized cell lines would be amenable to genetic modification, which facilitates the dissection at the molecular level of immune functions and cell–pathogen interactions (i.e., replication cycle, host immune modulation, and pathogenesis), and represent valuable tools for the development of specific diagnostic assays, antiviral drugs, and vaccine candidates.

Weingartl et al. generated three cell lines (designated 3D4/2, 3D4/21 and 3D4/31), available from the American Type Culture Collection (ATCC Number: CRL-2843, CRL-2844, and CRL-2845), by transfecting primary porcine PAM with the pSV3-neo plasmid, which carries genes for neomycin resistance and the SV40 large T antigen (SV40LT) [280]. These cell lines express the pan-myeloid marker CD172a and support the replication of several porcine viruses, such as ASFV, CSFV, PRV, vesicular stomatitis virus, swine vesicular disease virus or swine poxvirus. However, these cell lines lack the expression of CD163, CD169 and other macrophage markers and fail to support the replication of PRRSV, suggesting that they do not possess all the features of PAM [281,282,283].

Zuckermann et al. derived a pig macrophage cell line, named ZMAC-4, from fetal pig lung macrophages. ZMAC-4 cells, like PAM, express CD14 and CD172a and the transcription factor PU.1. They also express CD163, but at lower levels than PAM, and are negative for CD203a [284,285]. These cells are phagocytic and produce IFN-α in response to poly-I:C with similar kinetics as PAM, and support the replication of PRRSV and ASFV, representing an alternative to primary porcine macrophage cultures for cell–virus interaction studies, virus titration and detection, and for vaccine production.

Primary PAM cells were transduced by Sansong et al. with the human telomerase reverse transcriptase (TERT) using a retrovirus vector [286]. The exogenous expression of TERT protein restores telomerase activity and allows cells to proliferate indefinitely. Four immortalized PAM clones were established that expressed high levels of porcine CD163 and CD169 on the plasma membrane cell surface and efficiently support the replication of both type 1 and type 2 PRRSV isolates, as well as of other viruses that replicate in porcine macrophages such as PRV, CSFV and porcine circovirus 2 (PCV2).

Similarly, Takenouchi et al. established an immortalized cell line by transferring the SV40 large T antigen and porcine TERT genes into primary porcine kidney-derived macrophages using lentiviral vectors [287]. Using the same approach, these authors also successfully immortalized macrophages from lung and small intestine [288,289]. These cells retain many features of primary macrophages and are susceptible to infection by ASFV or PRRSV and may represent useful tools for studying the interaction of these porcine pathogens with macrophages [288,290,291]. 

More recently, Meek et al. have described a strategy to derive porcine macrophages from stem cells using a three-phase protocol, adapted from a method devised for mouse and human pluripotent stem cells (PSCs) [292]. These porcine PSC-derived macrophages (PSCdMs) exhibited molecular and functional characteristics of ex vivo primary macrophages and were productively infected by a variety of porcine pathogens, including PRRSV and ASFV. They expressed the macrophage markers CD172a, CD14, CD16, CD163 and CD169; displayed a transcriptional profile similar to PAM; and were highly phagocytic. The feasibility of the genetic engineering of these cells affords new opportunities for studying host–pathogen interactions and genes associated with innate immunity in swine.

## 5. Conclusions

Macrophages are present in all organs, where they perform essential functions in host defense and the maintenance of tissue homeostasis. They are highly plastic cells, which in response to signals from their surrounding micro-environment, can reprogram their gene transcription profile, displaying a broad variety of phenotypes and functions. In swine, macrophages are also primary targets for the replication of several economically relevant pathogens, such as ASFV, CSFV or PRRSV, and play a determining role in the pathogenesis of the diseases caused by them.

All this has spurred the study of these cells in the pig in the past decade, leading to important advances in the characterization of receptors expressed on their surface, with a considerable increase in the toolbox available for their analysis. These reagents allow for obtaining insight into the heterogeneity and plasticity of macrophages within the different tissue compartments and, together with high throughput genomic and proteomic techniques, will contribute to a better understanding of their specific functions as well as their involvement in disease pathogenesis, which will enable the design of novel strategies to control them.

The advances in our knowledge of the biology of porcine macrophages are also of interest for research in different human inflammatory diseases and for the search of new therapeutic targets to treat them, given the increasing use of pig as a model for biomedical research, supported by the similarities between pigs and humans and the advances in the annotation of pig genome and in cell-based transgenic techniques.

## Figures and Tables

**Table 1:** Available monoclonal antibodies to study porcine macrophages. Selection is based on molecules described in the text.

Specificity	Clone Name	Reference	Supplier
CD11b	MIL4	[10,11]	Bio-Rad/Serotec
CD11c	3A8	[12]	Bio-Rad/Serotec
CD14	MIL2	[10]	Bio-Rad/Serotec
CD16	G7	[13]	BD Biosciences Bio-Rad/Serotec
CD32	HuCAL32 (CD32a), HuCAL 91 (CD32a/b)	[14]	
CD32a/b	AT-10	[15]	Bio-Rad Thermo Fisher
CD68	EBM11	[16]	Dako
CD107a	4E9/11	[8,17]	Bio-Rad/Serotec
CD115	ROS8G11	[18]	Bio-Rad/Serotec
CD163	2A10/11	[19]	Bio-Rad/Serotec
CD172a	74-22-15 BL1H7	[20] [21]	ATCC/Kingfisher Biotech Bio-Rad/Serotec
CD200R1	PCT1 and PCT3	[22]	
CD200R1L	PCT1	[22]	
CD203a (ENPP1)	PM18-7	[23]	Bio-Rad/Serotec
CD205	ZH9F7	[24]	Bio-Rad/Serotec
CD206 (MR)	122D2.08	[25]	Dendritics
CD209	DC428	[26]	
CLEC12A (CD371)	FA2B10	[27]	
CLEC12B	PELE6	[28]	
F4/80 (ADGRE1)	ROS-4E12-3E6	[29]	
Siglec-1 (CD169)	3B11	[30]	Bio-Rad/Serotec
Siglec-3 (CD33)	5D5	[31]	Bio-Rad/Serotec
Siglec-5 (CD170)	4F7	[32]	Bio-Rad/Serotec
Siglec-10	2E9	[33]	Bio-Rad/Serotec
TLR2 (CD282)	1H11	[34]	Bio-Rad/Serotec
TLR3 (CD283)	TLR3.7	[9,35]	eBioscience/Thermofisher
TLR4 (CD284)	3H3	[36]	Bio-Rad/Serotec
TLR9 (CD289)	26C593.2 eB72-1665	[37] [35]	Novus Biologicals eBioscience

**Table 2:** Macrophage subsets in different organs.

Tissue	Location	Phenotype	Comments	Ref.
Spleen				
	red pulp	CD163^+^ CD169^−/lo^	APC in vitro	[30,121,152]
	marginal zone and ellipsoids	CD163^−^ CD169^+^	APC in vitro	
	follicles white pulp	CD107a^hi^	Tingible body MØ	[17]
Lymph nodes				
	subcapsular area/periphery of LN	CD163^+^ CD169^+^	Equivalent to mouse medullary sinus macrophages	[30,121,247,248]
	perifollicular zone and inside B cell follicles	CD163^−^ CD169^+^	Equivalent to murine subcapsular sinus macrophages	
	medullary cords	CD163^+^ CD169^−^	Equivalent to murine medullary cord	
	follicles	CD107a^hi^	Tingible body MØ	[17]
Tonsil				
	crypt, epithelium, connective tissue and follicles and interfollicular region	CD172a^hi^ CD14^−^ (Tuk4) CD163^+^ MHC-II^hi^		[249]
Liver				
	hepatic sinusoids	CD107a^+^ CD163^+^ CD169^+^	Kupffer cells	[17]
Lung				
	alveolar macrophages (AM)	CD163^+^ CD169^+^ CD172a^+^ CD203a^+^ CD206^+^	Suggested embryonic-derived macrophages. Role in protection against influenza virus infection Maintaining airway immune homeostasis.	[170,206,219,250,251]
	lung parenchyma (PIM)	Like AM	Suggested embryonic-derived macrophages.	[219,252,253]
	lung parenchyma moMacro	CD172a^int^CD163^int^	Monocyte-derived cell characteristics,	[170]
Nasal mucosa				
	upper lamina propria and epithelium	CD163^+^ CD169^−^		[254,255]
	deep lamina propria, close to cartilage	CD163^+^ CD169^+^		
Skin				
	Dermis	CD172a^+^CD163^+^SLA-DR^−/lo^ CD14^+^CD16^+^	Poor T cell stimulatory capacity	[25,169]
Placenta				
	fetal placenta and endometrium,	CD163^+^ CD169^+^	Absent during mid gestation in fetal placenta	[256,257]
	fetal placental and endometrium,	CD163^+^ CD169^−^		

## Data Availability

Not applicable.
